# Multiresidue analysis of bat guano using GC-MS/MS

**DOI:** 10.1007/s00216-024-05263-3

**Published:** 2024-04-02

**Authors:** Michelle Peter, Nikita Bakanov, Xenia Mathgen, Carsten A. Brühl, Michael Veith, Christoph Müller

**Affiliations:** 1 Department of Pharmacy, Center for Drug Research, Ludwig-Maximilians-Universität München, 81377 Munich, Germany; 2 iES Landau, Institute of Enivonmental Sciences Landau, University Kaiserslautern-Landau, 76829 Landau, Germany; 3 Department of Biogeography, Trier University, 54296 Trier, Germany; 4 State Office for Agriculture and Environement of Western Pomerania, 18439 Stralsund, Germany

**Keywords:** Chiroptera, Contamination monitoring, Fenazaquin, Multi-walled carbon nanotubes (MWCNTs), QuEChERS

## Abstract

Bats are the second largest mammalian order and are an endangered species group with a strong need for contamination monitoring. To facilitate non-invasive monitoring of the ecological burden in bat populations, a multiresidue method for the simultaneous quantification of 119 analytes including pesticides, persistent organic pollutants (POPs), active pharmaceutical ingredients (APIs), polycyclic aromatic hydrocarbons (PAHs), UV blockers, plasticizers, and other emerging pollutants in bat guano with gas chromatography tandem mass spectrometry (GC-MS/MS) was developed. Sample preparation and clean-up were performed with a modified QuEChERS approach based on DIN EN 15662. The method uses 1.00 g bat guano as sample with acetonitrile and water for liquid-liquid extraction. Phase separation is assisted by citrate-buffered salting out agent. For clean-up of the extract, primary secondary amine (PSA) was combined with graphitized carbon black (GCB). The lower limits of quantification (LLOQ) ranged between 2.5 and 250 µg kg^−1^. Linearity was shown in a concentration range from the respective LLOQs to 1250 µg kg^−1^. The median of the mean recovery was 102.4%. Precision was tested at three concentrations. Method and injection precision were adequate with a relative standard deviation (RSD) below 20%. Furthermore, the comparative analysis with LC-MS/MS demonstrated the reliability of the results and provided a valuable extension of the analytical scope. As proof of concept, three guano samples from a German nursery roost of *Myotis myotis* were analysed. The results show a time-dependent change in contaminant concentration, highlighting the strong need for non-invasive contamination monitoring of whole bat populations.

**Graphical Abstract:**

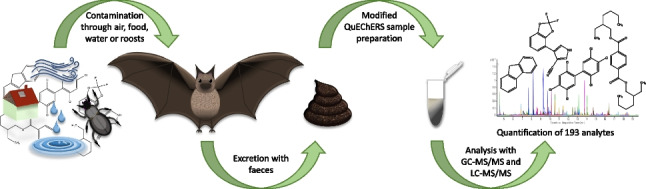

**Supplementary Information:**

The online version contains supplementary material available at 10.1007/s00216-024-05263-3.

## Introduction

Bats are mammals of the order Chiroptera, which includes over 1400 classified species, making it the second largest mammalian order after rodents [1]. Due to their feeding habits, bats fulfil several important ecosystem functions. The species, which feed on nectar and pollen, serve as pollinators and help disperse seeds [2]. A large proportion of insectivorous bats feed on agricultural pests, contributing to biological pest control, valuable for farmers [3].

In Germany, 24 bat species are native; most of them are considered endangered [4]. Bat populations have declined dramatically worldwide over the past decades. Many possible causes have been reported, including direct human disturbance of roosts [5], deforestation and ecosystem destruction [2], water pollution [6, 7], increased construction of wind energy facilities [8], climate change [9], white-nose syndrome [10, 11], and increasing contamination and pesticide use [12, 13]. Exposure to pesticides has been shown to increase mortality in bats [14–16] and cause sublethal effects such as immunosuppression, impairment of the endocrine system, or reproductive disorders [17].

Due to their position in the food chain, bats are highly susceptible to bioaccumulation and biomagnification of pesticides and persistent organic pollutants (POPs) [18]. During their long lifespan of up to 41 years (*Myotis brandtii* (Brandt’s bat)) [19], they are continuously exposed to contaminants through food and water consumption or skin contact with hazardous vapours [17]. The predominantly lipophilic substances accumulate in stored body fats. By mobilising these reserves during hibernation, migration, or lactation, pesticide concentrations can reach peak levels, e.g. in the brain, which can be lethal [20]. Increased bat mortality can lead directly to population decline, as reproductive rates are very low, with only one or two offspring per year [21, 22]. Bats are considered promising indicator species in many areas, including farming practice and bioaccumulation of pollutants, as they react very sensitive to environmental changes [18].

Besides pesticides and POPs, many emerging environmental pollutants are potential threats to nature and wildlife. Polycyclic aromatic hydrocarbons (PAHs, e.g. fluoranthene), for example, are found ubiquitous in the environment and have known carcinogenic and mutagenic effects on humans [23]. Residues of PAHs have been found in bat carcasses [24]. Other substances with high abundance and environmental toxicity are polybrominated diphenyl ethers (PBDEs), such as 2,2′,4,4′-tetrabromodiphenyl ether (BDE 47), that are used as flame retardants [25]. PBDEs are prone to bioaccumulation and have been shown to cause adverse effects on DNA stability, mitochondrial damage, and cytotoxicity [26]. As substances with a wide, dispersive use and high aggregated tonnage [27], plasticizers are of interest for pollution monitoring and risk assessment. Bis(2-propylheptyl) phthalate (DPHP), for example, is currently assessed by the European Chemicals Agency (ECHA) regarding its endocrine disruptive potential as well as risks originating from the exposure of sensitive populations [27]. Active pharmaceutical ingredients (APIs) were already found in wastewater, sewage sludge, and even drinking water [28, 29]. APIs that were found in water samples and sewage treatment plants include analgesics like acetaminophen [30], anticonvulsants (e.g. carbamazepine) [28], and sexual hormones like 17α-ethinylestradiol [29]. APIs can pose a high risk for adverse effects on nontarget wildlife through long-term exposure as well as synergistic effects of mixtures of substances [29, 31].

Close contamination monitoring in bat populations is hence essential. This task requires analytical methods that can quantify multiple residues with different physicochemical properties simultaneously. Currently applied multiresidue methods mainly analyse pesticides and POPs in either bat muscles [32], liver [33], whole carcasses [24], or hair [34]. This allows specific characterisation of the contamination of individual bats and can be of interest when deceased bats are collected. However, as these methods are invasive and require deceased or in case of hair analysis captivated [35] specimens, their scope is limited, as specific permits to handle protected species are required. A promising matrix for non-invasive contamination monitoring of bats is their guano. It is available in large quantities and can be collected with minimal disturbance to bat colonies. Guano allows monitoring of whole populations rather than individuals, and samples can be collected over any period of time to monitor changes in contamination [36, 37]. The faeces of insectivorous bats such as *Myotis myotis* consist mainly of fine particles of insect exoskeletons and thus of chitin and contain several mineral elements such as carbon, nitrogen, sulphur, and phosphorus; when fresh, as in our case, it is slightly acidic to near neutral (pH 5.1 to 7.3) [38].

Currently available methods for the contamination monitoring of bat guano are often centred on only few, partly outdated pesticides, mainly organochlorines and POPs [12, 37, 39] or organophosphates [6, 40] that are no longer applied. Assessing only a small subset of closely related compounds, their analytical scope is limited. Perfluoroalkyl substances (PFAS) have been assessed besides some parabens, benzophenones, and plasticizers with a LC-MS/MS method, covering 20 pollutants [41].

Here, we present a multiresidue method for the analysis of bat guano, which covers 57 pesticides (24 fungicides, 15 insecticides, 3 acaricides, and 12 herbicides as well as 1 biocide and 2 metabolites), 17 POPs, 17 APIs (including 2 hormones), 8 UV blockers, 5 polycyclic aromatic hydrocarbons (PAHs), 3 plasticizers, and 12 other emerging pollutants (4 fragrances, 2 antioxidants, 2 flame retardants, 2 indicators for human pollution, and 2 industrial chemicals). The presented method is, to our best knowledge, the first multiresidue method for bat guano based on gas chromatography tandem mass spectrometry (GC-MS/MS) that covers such a broad analytical scope. The method is based on the QuEChERS sample preparation concept introduced by Anastassiades *et al.* in 2003 [42]. DIN EN 15662 [43], which defines the standard methods for pesticide quantification in food and feed matrices in Europe, was used as a starting point for method development. The final method was validated for parallel analysis of 119 analytes according to SANTE 11312/2021 [44] regarding lower limit of quantification (LLOQ), linearity, recovery, and precision. As a proof of concept, three guano samples from a German nursery roost of *Myotis myotis*, the greater mouse-eared bat, were analysed with the newly developed method. The same samples were also analysed with high-performance liquid chromatography (LC)-MS/MS for 97 current use pesticides (37 fungicides, 36 herbicides, 21 insecticides, and 3 biocides) [45], following the principle of QuEChERSER (“more than QuEChERS”) [46], broadening the analytical scope, as the implementation of both LC and GC enables the determination of more analytes than the utilization of only one chromatographic system would [46].

## Experimental

### Chemicals

All analytes and the deuterated internal standards azoxystrobin-*d*_4_, chlorpyrifos-*d*_10_, and pyrene-*d*_10_ (all >98%) were purchased from BLDpharm (Kaiserslautern, Germany), ChemPUR (Karlsruhe, Germany), EDQM (Strasbourg, France), HPC Standards GmbH (Cunnersdorf, Germany), Merck (Darmstadt, Germany), or Tokyo Chemical Industries (TCI, Tokyo, Japan).

Acetonitrile (ACN) in HPLC grade was purchased from VWR (Darmstadt, Germany). 3-Ethoxy-1,2-propanediol (98%) was obtained from BLDpharm (Kaiserslautern, Germany). L-Gluconic acid γ-lactone (95%), D-sorbitol (99%), sodium citrate dihydrate (≥99%), and disodium hydrogen citrate sesquihydrate (≥99%) were purchased from Merck (Darmstadt, Germany). Shikimic acid (≥98%) was purchased from Carl Roth (Karlsruhe, Germany). SPE bulk sorbents of ethylenediamine-*N*-propyl-functionalized silica (PSA), octadecyl functionalized silica (C18), and graphitized carbon black (GCB) were obtained from Agilent Technologies (Santa Clara, CA, USA). The bulk of multi-walled carbon nanotubes (MWCNT, 95%) with an outside diameter of 20–30 nm was obtained from abcr (Karlsruhe, Germany). Anhydrous magnesium sulphate (MgSO_4_, ≥98%) was purchased from Grüssing (Filsum, Germany). Sodium chloride (NaCl, p.a.) was obtained from Bernd Kraft (Duisburg, Germany).

### Reagents and starting materials

#### Guano bulk and samples

Guano samples as well as a bulk of guano for method development were collected in an urban nursery roost of *Myotis myotis* in Ahrbrück (Germany). Sampling occurred from May to July 2019. Samples were stored at −80 °C until analysis.

#### Stock solutions and analyte protectants

Stock solutions of all analytes and the three internal standards (azoxystrobin-*d*_4_, chlorpyrifos-*d*_10_, and pyrene-*d*_10_) were prepared with a concentration of 1.00 mg mL^−1^. The analyte stock solutions were combined into five groups according to their chemical properties (amines/amides, acids, nonpolar compounds, alcohols, and esters/ethers) and diluted to five different working solutions (0.01 mg mL^−1^) with ACN. An internal standard working solution containing azoxystrobin-*d*_4_, chlorpyrifos-*d*_10_, and pyrene-*d*_10_ (5.0 µg mL^−1^) was prepared accordingly. All stock and working solutions were stored at −20 °C and allowed to adjust temperature for 1 h at room temperature before use.

The analyte protectants were prepared from 3-ethoxy-1,2-propanediol (200 mg mL^−1^), L-gluconic acid γ-lactone (10 mg mL^−1^), shikimic acid (5 mg mL^−1^), and D-sorbitol (5 mg mL^−1^) in a mixture of ACN and water 6:4 (*v/v*) according to an application note from the EU Reference Laboratories for Residues of Pesticides (EURL) [47] and stored at 8 °C.

#### Buffer salt and dSPE

The buffer salt mixture was prepared according to DIN EN 15662 [43]. For the prospective sample size of 1.00 g (±0.01 g) guano, a total amount of 3.25 g (±0.05 g) buffer salt mixture was used. The mixture consisted of anhydrous magnesium sulphate, sodium chloride, trisodium citrate dihydrate, and disodium monohydrogen citrate sesquihydrate in a ratio of 8:2:2:1.

For final sample preparation, the dSPE mixture was prepared from anhydrous magnesium sulphate, ethylenediamine-*N*-propyl-functionalized silica (PSA), and graphitized carbon black (GCB) in a ratio of 60:10:3. A portion of 91 mg (±1 mg) dSPE mixture was used per 0.5 mL sample extract.

#### Matrix characterisation

Air-dried guano samples and bulk were ground to a fine powder with a mortar and pestle prior to extraction. Particle size distribution was assessed according to Ph. Eur. 2.9.38 [48] with an AS 200 system (Retsch, Haan, Germany). Sieve mesh sizes ranged from 2500 to 125 µm. Samples were sieved with an amplitude of 50 mm for 15 min.

Water content was determined using two different approaches, in hexaplicates. For determination *via* loss on drying [49], 2.00 g (±0.10 g) of ground guano was weighed into watch glasses and dried in a drying cabinet (Binder, Tuttlingen, Germany) at 80 °C until constant mass for 20 h ± 1 h. Karl-Fischer titration was performed with a Titrator Compact 20VS (Mettler Toledo, Columbus, OH, USA), using 50 mg (±5 mg) bat guano.

### Instruments

#### Sample preparation

All shaking steps were performed using a Vortex Genie from Scientific Industries (Bohemia, NY, USA). For centrifugation of 15 mL tubes, an EBA 20 centrifuge, Hettich (Tuttlingen, Germany) was used. All microcentrifuge tubes were centrifuged with a 5415 D centrifuge from Eppendorf (Hamburg, Germany).

#### Gas chromatography tandem mass spectrometry

Samples were analysed using a GC-MS/MS system consisting of a 7890B gas chromatograph coupled with a 7010B triple quadrupole mass spectrometer with a high-efficiency source (HES), both from Agilent (Santa Clara, CA, USA). The chromatograph was equipped with two Agilent J&W HP 5MS ultra inert capillary columns (15 m × 250 µm × 0.25 µm) coupled by a capillary flow technology (CFT) backflush device. The multi-mode inlet (Agilent) was operated in solvent vent mode. For injection, a PAL3 RSI autosampler from CTC analytics (Zwingen, Switzerland) was used. All parameters of the GC-MS/MS acquisition method are given in Table 1. Data were acquired operating the MS/MS in dynamic multiple reaction monitoring (dMRM). The method parameters for each analyte can be found in Electronic Supplementary Material (ESM) Table S1.

**Table 1:** Parameters of the GC and the MS part of the developed method

GC parameters	
Injection volume	1 µL
Split ratio	Splitless
Inlet starting temperature	60 °C (hold for 0.2 min)
Inlet temperature ramp	900 °C min^−1^, 60–280 °C (hold for 20.75 min) During post run to 310 °C
Oven temperature	60 °C (hold for 1 min) 40 °C min^−1^, 60–170 °C 10 °C min^−^^1^, 170–310 °C (hold for 3 min)
Carrier gas	Helium 5.0 (Air Liquide, Düsseldorf, Germany)
Carrier gas flow rate	1.1 mL min^−1^ on column 1 1.3 mL min^−1^ on column 2
Back flush flow rate	−4.0 mL min^−1^ on column 1 4.4 mL min^−1^ on column 2
Vent flow	100 mL min^−1^
Run time	20.75 min
Post run time	5 min
Cycle time	31 min
MS parameters	
Transfer line temperature	280 °C
Ion source	EI, positive
Ion source voltage	70 eV
Ion source temperature	230 °C
Quadrupole temperature	150 °C
Collision gas	Argon 4.5 (Air Liquide, Düsseldorf, Germany)
Collision gas flow	0.9 mL min^−1^
Quench gas	Helium 5.0 (Air Liquide, Düsseldorf, Germany)
Quench gas flow	2.25 mL min^−1^
Solvent delay	4 min

### Final sample preparation

For the final sample preparation, 1.00 g (±0.01 g) ground guano was weighed into 15 mL centrifuge tubes and three stainless steel beads were added. After addition of 10 µL of the internal standard working solution (5.0 µg mL^**−**1^ of azoxystrobin-*d*_4_, chlorpyrifos-*d*_10_, and pyrene-*d*_10_), samples were extracted with 5.0 mL water and 5.0 mL ACN. The mixture was vortexed for 30 s, and after 15 min resting time, 3.25 g (±0.05 g) buffer salt mixture was added and immediately shaken by hand to prevent the formation of agglomerates. The mixture was vortexed a second time for 30 s and afterwards centrifuged for 5 min at 3400 *g* at room temperature. After centrifugation, 0.5 mL of the upper organic layer was transferred to a microcentrifuge tube containing 91 mg (±1 mg) of dSPE mixture and immediately shaken by hand. After vortexing for 30 s, the tubes were centrifuged for 5 min at 12,000 *g* at room temperature. One hundred microliters of the supernatant was transferred to a GC glass vial with insert and 3 µL of analyte protectants was added. The sample was vortexed for 30 s and then analysed with GC-MS/MS. For quantification, procedural calibration standards were used and measured in a bracketing manner.

### Method validation

Method validation was performed according to SANTE/11312/2021 [44] regarding linearity, LLOQ, recovery, and precision. Validation experiments were performed with all 119 analytes in hexaplicates. For the determination of linearity, guano extract was spiked with ten levels (2.5 µg kg^**−**1^, 5.0 µg kg^**−**1^, 10 µg kg^**−**1^, 50 µg kg^**−**1^, 100 µg kg^**−**1^, 250 µg kg^**−**1^, 500 µg kg^**−**1^, 750 µg kg^**−**1^, 1000 µg kg^**−**1^, and 1250 µg kg^**−**1^) of each analyte. To assess recovery, one set of samples was spiked before and another set after sample preparation. Recovery was assessed at least at three concentrations (*n*=6) for LLOQs ≥ 250 µg kg^**−**1^, at four concentrations for analytes with a LLOQ of 100 µg kg^**−**1^ and at five concentrations for analytes with LLOQs < 100 µg kg^**−**1^. Precision was evaluated as method precision and as injection precision. Samples were spiked at the respective LLOQ of each analyte as well as 500 µg kg^**−**1^ and 1000 µg kg^**−**1^.

### Quantification

Quantification was performed with matrix-matched procedural calibration standards. Blank matrix samples were spiked prior to extraction with all 119 analytes at concentrations from their respective LLOQ to 1250 µg kg^**−**1^ or 5000 µg kg^**−**1^ and afterwards processed as described above. Calibration standards were prepared and analysed in a bracketing manner in the same batch as the samples. The application of matrix-matched procedural calibration standards enables the reduction of method bias due to low clean-up yields as well as for compensating matrix effects according to SANTE/11312/2021 [44]. In accordance with SANTE/11312/2021 [44], the limit of detection (LOD) levels were not determined. Analytes were considered as detected (not quantified, n.q.), if all three MRM transitions were present in the chromatogram at the retention time range of the analyte.

### LC-MS/MS analysis

The guano samples were analysed with LC-MS/MS according to a published methodology [45], originally intended to analyse 97 currently used pesticides in soil and herbaceous vegetation [45]. Prior to analysis, the raw extract was cleaned up with a dSPE mixture containing 150 mg (±5 mg) anhydrous magnesium sulphate and 100 mg (±2 mg) PSA per mL extract.

## Results and discussion

### Matrix characterisation

Prior to method optimization, the matrix was characterised regarding particle size and water content and the extraction solvent ratio was adapted accordingly. Ensuring a uniform particle size distribution of the processed guano samples is crucial for obtaining reproducible results and minimizing variations in extraction efficiency. The particle size analysis of the ground guano matrix showed that 89% of all particles were smaller than 500 µm, about 10% were smaller than 900 µm, and only less than 1% were larger than 900 µm. The applied processing method with a mortar and pestle was feasible and provided a finely ground powder with sufficiently small particles.

According to the DIN EN 15662 [43], ACN and water were chosen as extraction solvents. As the water content of the guano matrix was determined to be about 5% (5.4% by loss on drying, 5.1% by Karl-Fischer titration), a sample size of 1.00 g guano required 5.0 mL of ACN and 5.0 mL of water for liquid-liquid extraction, in line with the specifications of the DIN EN 15662 [43] for matrices with very low water content (<15%) [43]. For the following salting out step, a citrate-buffered salt mixture according to DIN EN 15662 [43], containing anhydrous magnesium sulphate, sodium chloride, trisodium citrate dihydrate, and disodium citrate sesquihydrate in a ratio of 8:2:2:1, was used. The applied amount of the salt mixture was adjusted to the sample size of 1.00 g and the applied amount of water, so 3.25 g salt mixture was used.

### Method optimization

To facilitate the issue of non-invasive contamination monitoring of bats, a multiresidue method capable to analyse a broad range of analytes with different physicochemical properties at the same time was developed. For this challenging analytical scope, the DIN EN 15662 [43] was used as a starting point for method optimization.

In the first step, the ideal composition of dSPE mixture was evaluated, followed by the determination of matrix effects and the influence of analyte protectants. For all method optimisation steps, all 119 analytes were considered at three levels (50 µg kg^**−**1^, 250 µg kg^**−**1^, and 1000 µg kg^**−**1^).

#### Dispersive solid-phase extraction

For achieving the most effective clean-up of the raw sample extract, four different sorbents were tested regarding their effect on the measured peak area of all analytes. Blank bulk samples were spiked with all analytes prior to the clean-up step at the concentrations of 50 µg kg^−1^, 250 µg kg^**−**1^ and 1000 µg kg^**−**1^. Experiments were performed in triplicates. Evaluation of the sorbents was based on the obtained peak area of all analytes after clean-up as well as standard deviation. The average peak areas were calculated by adding the peak area sums of all analytes per sample and calculating the mean (*n*=3) and standard deviation per applied sorbent or sorbent mixture, resulting in one value per tested clean-up.

**Fig. 1:**
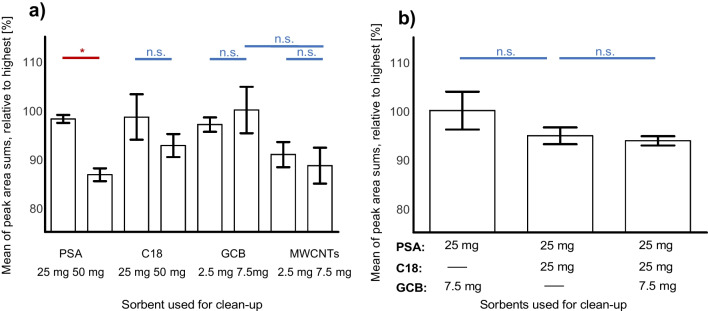
Peak area sums over all analytes obtained after clean-up with different dSPE sorbents (**a**) and dSPE sorbent mixtures (**b**), relative to the highest result (*n*=3, error bars equal standard deviation); lines indicate significance (red, *) or non-significance (blue, n.s.) (two-tailed *t*-test, CL=95%); sorbent amount given in mg, used for clean-up of 1 mL extract

PSA, C18, and GCB are well-established sorbents for dSPE, featured in the DIN EN 15662 [43] for the clean-up of different matrices. Multi-walled carbon nanotubes (MWCNTs) have more recently been applied in sample clean-up as a promising sorbent and potential alternative for GCB, removing mainly planar substances like pigments or sterols from the matrix [50–52]. After testing the individual sorbents regarding their impact on peak areas and standard deviations, they were combined to mixtures containing PSA with either GCB, C18, or both. The application of 7.5 mg MWCNTs led to notable lower peak area sums than the same amount of GCB (see Fig. 1a), negatively affecting planar analytes like fluoranthene or pyrene, so they were not considered for further application. For further improvement, the sorbents providing the highest peak area sums (25 mg PSA, 25 mg C18, and 7.5 mg GCB) were chosen. As the application of 25 mg PSA also performed best regarding standard deviation, it was combined with 25 mg C18, or 7.5 mg GCB or both, respectively.

From the tested mixtures, the combination of 25 mg PSA with 7.5 mg GCB per mL raw extract provided the best results regarding peak area sum (Fig. 1b). The addition of C18 leads to lower peak areas; therefore, the mixture of 25 mg PSA with 7.5 mg GCB was used for further optimization.

To evaluate the ideal ratio of raw extract to sorbent, the amount of the sorbent mixture (both PSA and GCB) that was used to clean up 1 mL extract was doubled and halved whilst the amount of anhydrous magnesium sulphate stayed constant (150 mg). The more sorbent used for clean-up, the lower the standard deviation and peak area sums were. As a result, the combination of 25 mg PSA and 7.5 mg GCB per mL raw extract provided the best compromise between high peak areas and a low standard deviation. The final processing step was scaled down by a factor of two in order to decrease the amount of sorbents used.

#### Matrix effects and analyte protectants

The SANTE/11312/2021 guideline [44] defines matrix effects as “an influence of one or more co-extracted compounds from the sample on the measurement of the analyte concentration or mass. It may be observed as increased or decreased detector response compared with that produced by solvent solutions of the analyte”.

Within GC-MS/MS measurements, matrix effects occur as matrix-induced response enhancement and are a well-known phenomenon. Matrix components may mask active sites in the liner and column, increasing analyte transfer to the analyser [53–55]. This effect can result in different measured peak areas in matrix compared to solvent samples of the same actual concentration. It therefore may affect quantification if solvent samples are used as calibration standards. Hence matrix effects beyond 20% have to be addressed during method development, according to SANTE/11312/2021 [44].

To determine the matrix effects in the guano samples, all analytes were spiked into guano extract and pure solvent (ACN) at 250 µg kg^**−**1^. Each sample was measured 10 times, and the mean area was used to calculate matrix effects according to the following Formula 1:

1 $$\left(\frac{{{\text{mean}}}_{\mathrm{spiked\;matrix}} -{\mathrm{ mean}}_{\mathrm{spiked\;ACN}}}{{{\text{mean}}}_{\mathrm{spiked\;ACN}}}\right)\times 100$$

**Fig. 2:**
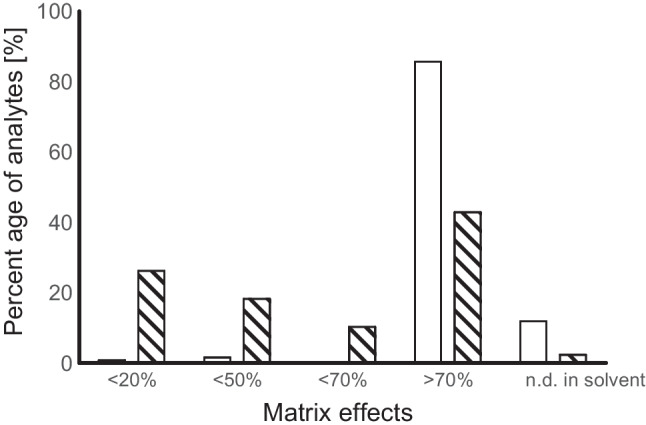
Percentage distribution of analytes affected by matrix effects; without (blank column) and with (striped column) the addition of analyte protectants; n.d.: not detected in pure solvent samples

Figure 2 shows the percentage distribution of matrix effects. As some of the analytes were not detectable (n.d.) in the spiked solvent samples but only in the matrix-matched samples (marked as n.d. in solvent, Fig. 2), matrix effects could not be calculated for 12% of the analytes. Besides, all other analytes showed response enhancement in matrix, with increased signals up to nearly 7000% for Uvinul A plus. In total, over 85% of analytes showed very high (>70%) matrix effects.

An established possibility to compensate matrix effects in GC analysis is the addition of analyte protectants, like D-sorbitol, L-gluconic acid γ-lactone, or 3-ethoxy-1,2-propanediol, in excess, to both, the spiked solvent sample as well as the matrix-matched sample. Analyte protectants can have the same effect on analytes as the matrix components [47]. They can interact with the active sites in liner and column, masking them, increasing the amount of analytes reaching the detector and subsequently the detector response. The addition of analyte protectants to both, matrix-matched and solvent samples, in excess, is thus a possibility to equalize the detector responses of the analytes in both samples.

Figure 2 shows that the addition of analyte protectants could reduce the number of analytes affected by very high matrix effects from previously over 85 to 43%. Analyte protectants helped compensate the matrix effects to a certain extent. However, over 70% of all analytes still showed matrix effects above 20%. As few analytes (2.4%) were not detectable in spiked solvent samples, the use of matrix-matched calibrants was inevitable and combined with the addition of analyte protectants to both, the external matrix-matched calibration, and the samples.

**Fig. 3:**
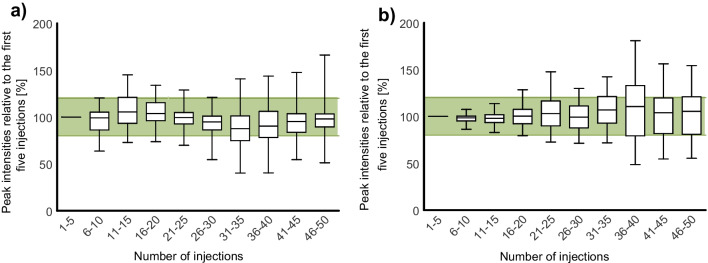
Effect of multiple, subsequent injections on analyte peak area measured in cleaned-up matrix extract without (**a**) and with (**b**) the addition of analyte protectants; mean after each five injections; green area represents acceptable discrepancy (±20%); boxes represent 50% of analytes, line indicates median, whiskers show minimum and maximum values

In addition, it was tested whether analyte protectants could contribute to a reliable, robust method by stabilizing analyte response over the course of a sequence. Figure 3 shows the mean response per analyte over 50 consecutive measurements of the same sample without (Fig. 3a) and with (Fig. 3b) added analyte protectants. When no analyte protectants were present in the sample, analyte peak areas started to drift after the first five injections, whereas the addition of analyte protectants helped to reduce the deviation of most analytes below 20% compared to the first five measurements over at least 20 injections. The increased scatter after injections 21 to 25 was resolved by implementing bracketing calibration.

### Method validation

The optimized method was validated for all analytes according to SANTE/11312/2021 [44]. All validation data are shown in ESM Table S2. Selectivity of the method was ensured through the dynamic multiple reaction monitoring (dMRM) applied, analysing three specific precursors to product ion transitions per analyte at a specific retention time. No interfering signals from endogenous components or other analytes were detected in the retention time range of the analyte. In accordance with SANTE/11312/2021 [44], the limit of detection (LOD) levels were not determined.

Most analytes showed linear responses in concentration ranges from their respective LLOQ (2.5 µg kg^**−**1^ to 250 µg kg^**−**1^) to 1250 µg kg^**−**1^. For fluoranthene and pyrene, a concentration range of 750 to 5000 µg kg^**−**1^ was selected, due to their high endogenous concentration in guano bulk that was used for method validation and calibration curves. The lowest level with a relative standard deviation (RSD) of ≤20%, which also met the criteria for recovery and precision, was chosen as LLOQ for each analyte.

Recovery was assessed at three to five levels, depending on the respective LLOQ of each analyte. The calculated concentrations of recovery samples were plotted against their actual concentration. The slope of the linear function equals the mean recovery. According to SANTE/11312/2021 [44], the recovery should be between 70 and 120%; however, a recovery in the range of 30–140% is acceptable if consistent (RSD ≤20%). Ninety-one percent of the analytes had a mean recovery in the range of 70 to 120%, while 5% showed a recovery below 70% and 4% above 120%. However, the analytes with a mean recovery outside the desired range (ESM Table S3) showed consistency, with RSD <20% at all levels used for recovery determination. Nevertheless, to mitigate the potential recovery effects, procedural calibration standards were used for quantification [44].

To evaluate the method precision, six samples were spiked with all analytes at three concentrations, the respective LLOQ, a medium concentration (500 µg kg^**−**1^ or 2000 µg kg^**−**1^, respectively), and a high concentration (1000 µg kg^**−**1^ or 3750 µg kg^**−**1^, respectively). The precision of the method is expressed as RSD and must not exceed 20%. At all concentrations tested, all analytes met this criterion. For LLOQ, 79% of the analytes showed RSD values below 15% with a mean RSD across all analytes of 11.7% and for the medium and high concentrations tested, over 80% of all analytes showed RSD values ≤10% with a mean across all analytes of 7.3 and 7.7%, respectively.

System precision was assessed by repeated injection (*n*=6) of the same sample containing all analytes at the same three levels as for method precision. For 90% of all analytes, RSD values were below 10% at LLOQ and below 5% at the two other tested levels. The average system precision was 5.9% at LLOQ, 3.2% at medium concentration, and 3.0% at high concentration.

### Method application

As a proof of concept, the validated method was applied to three bat guano samples, collected in a nursery roost of *Myotis myotis* in Ahrbrück (Germany), between May and July 2019. For quantification, procedural standards were used to compensate for potential recovery losses. Bulk guano was spiked with all analytes, to get calibration standards, at six levels in the range of respective LLOQ to 1250 µg kg^**−**1^ or 5000 µg kg^**−**1^ respectively. The procedural standards were processed in the same batch and the same way as the guano samples.

In total, 32 different analytes were detected in the bat guano samples, with a minimum of 22 and a maximum of 27 per sample. A full list containing all analytes that could be detected as well as the corresponding amounts and substance classes is given in Table 2. Some analytes could be detected in all samples, but just below or close to their corresponding LLOQ, representing either a chronic exposure through air, food, water, and dermal exposure, or exogenous contamination of the guano after excretion, for example through plasticizers in containers used for sample collection and storage. Among those analytes were three of the PAHs (acenaphthene, acenaphthylene, and fluorene), the industrial chemical diphenylamine, the POPs lindane and dichlorodiphenyldichloroethylene (DDE), the musk-like fragrances galaxolide and tonalid, the plasticizers di-2-ethylhexyl adipate (DEHA) and di-2-propylheptyl phthalate (DPHP), the flame retardants triphenyl phosphate and a tetrabromodiphenyl ether (BDE 47), and the antioxidant butylated hydroxytoluene (BHT). Most of these analytes are either used in very high amounts in various industrial applications, e.g. BHT as antioxidant in gasolines, insulation oils, paints, plastic, or food and feed [56] as well as the plasticizer DPHP that is contained in polyvinyl chloride articles like cables, car interiors, PVC flooring, and medical devices and also in plastic toys [27, 57], or they are very persistent in the environment despite being prohibited years ago (e.g. lindane, DDE) [58]. It cannot be categorically ruled out that some of the analytes, especially the two fragrances and three plasticizers, originated from contaminations during or after sampling. As the sampling was carried out over 3 months in 2019, we are no longer able to trace the entire process. It could be possible that the sampling was carried out by staff with varying levels of training regarding sample handling. In the case of fragrances in particular, contamination by different perfumes or hand creams used on the day of sampling can lead to possible contamination. This can vary from sample to sample, depending on the date of and the person responsible for sampling.

**Table 2:** List of all analytes detected in the guano samples

Analyte	Substance class	May (µg kg^−1^)	June (µg kg^−1^)	July (µg kg^−1^)
Fenazaquin	Acaricide	n.q.	505	959
BHT	Antioxidant	n.q.	n.q.	n.q.
BDE 47	Flame retardant	n.q.	n.d.	n.q.
TPP	Flame retardant	n.q.	n.q.	n.q.
Galaxolid	Fragrance	n.q.	n.q.	n.q.
Tonalid	Fragrance	n.q.	n.q.	n.q.
Fenhexamid	Fungicide	n.d.	n.d.	n.q.
Fenpropimorph	Fungicide	n.d. n.d.*	n.q. 0.5*	n.q. 0.5*
Fludioxonil	Fungicide	n.d. n.d.*	n.d. n.d.*	85 67*
Fluopyram	Fungicide	n.d. n.d.*	n.d. n.d.*	n.q. 1*
Propiconazole	Fungicide	n.d.	n.q.	3
17β-Estradiol	Hormone	n.q.	n.d.	n.d.
Estrone	Hormone	n.q.	n.d.	n.d.
Testosterone	Hormone	127	220	308
Caffeine	Indicator for human pollution	12	n.d.	13
Diphenylamine	Industrial chemical	n.q.	n.q.	n.q.
Acenaphthene	PAH	n.d.	n.q.	n.q.
Acenaphthylene	PAH	n.q.	n.q.	n.q.
Fluoranthene	PAH	n.q.	n.q.	1120
Fluorene	PAH	n.q.	n.q.	n.q.
Pyrene	PAH	n.q.	807	1130
DEHA	Plasticizer	n.q.	n.q.	n.q.
DEHTP	Plasticizer	95	n.q.	60
DPHP	Plasticizer	n.q.	n.d.	n.q.
BHC β	POP	n.q.	n.d.	n.d.
BHC γ	POP	n.q.	n.q.	n.d.
DDE	POP	5	n.q.	n.q.
PCB 138	POP	27	30	35
PCB 153	POP	63	49	54
PCB 180	POP	34	30	30
EHS	UV blocker	n.q.	n.q.	n.q.
Octocrylene	UV blocker	n.q.	n.d.	n.d.

^*^Analysed with LC-MS/MS. n.d., not detected; n.q., not quantified (detected below LLOQ)

Fungicides like fenhexamid, fenpropimorph, and fluopyram were detected in concentrations below their respective LLOQ in at least one of the samples. The fungicides fludioxonil and propiconazole were quantifiable in the sample taken in July. The acaricide fenazaquin was detected in all three samples, with quite high concentrations of up to 958 µg kg^−1^ in July. In general, the concentrations of the pesticides tended to increase over the sampling period; hence, a conceivable origin of the pesticides is their application in agriculture during the growing season. Fenpropimorph and propiconazole are additionally used as wood preservatives [59] and may therefore originate from the roof truss in which the colony is located.

The polychlorinated biphenyls (PCBs) with a high chlorination degree (congeners 138, 153, and 180) were quantifiable, in comparable concentrations in all three samples. These results correspond with the prevailing contamination of bats with POPs like PCBs, DDE, or lindane that has previously been shown in bat liver samples, despite their prohibition decades ago [60].

Regarding the hormones detected, it is noticeable that only in the sample taken in May, 17β-estradiol and estrone were detectable, whereas the testosterone concentration increased over time and peaked in the sample from July. All three hormones, which are APIs, probably occur endogenously in bats.

Other analytes that could be quantified in the guano samples were the plasticizer di-2-ethylhexyl terephthalate (DEHTP), the UV blockers 2-ethylhexyl salicylate (EHS) and octocrylene, and caffeine.

Overall, the contamination of the guano samples increased over the sampling time. The sample taken in July contained 11 analytes at concentrations above their respective LLOQ, whereas in the samples from May and June only six or five analytes, respectively, were quantifiable. In addition, the detected concentrations of fenazaquin, testosterone, and pyrene were highest in the sample from July.

These results suggest a time-dependent change in the contamination of the guano samples. However, three samples are not sufficient to draw reliable conclusions about the actual burden in bat faeces. A study with several samples over a longer period of time is planned.

### Measurement with LC-MS/MS

The combined measurement of samples with GC- and LC-based methods provides a promising approach to broaden the measurable analytical spectrum, as the two approaches cover analytes with different physicochemical properties. To broaden the analytical scope, the guano samples were also analysed with an LC-MS/MS system with a previously published method, originally intended for analysis of 97 current used pesticides in soil and herbaceous vegetation [45], since no validated method for the analysis of guano with LC-MS/MS was available. As this method is not validated for analysis of guano samples, a method comparability experiment was conducted. Therefore, three of the analytes that were covered with both analytical methods, boscalid, fenpropimorph, and fludioxonil, were spiked into blank samples (100 µg kg^**−**1^ each) and processed as stated in the method section.

**Fig. 4:**
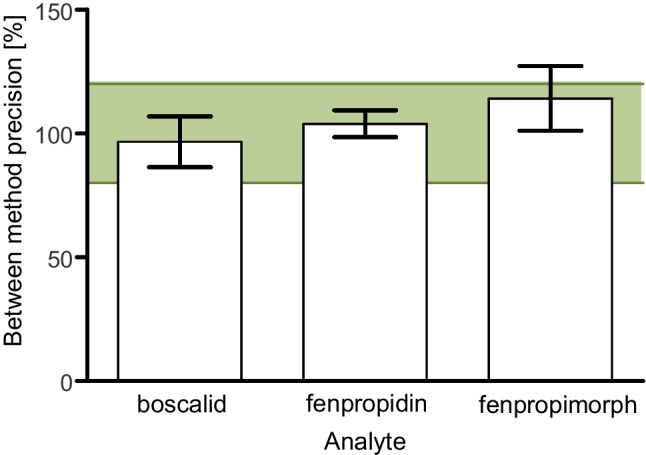
Method comparison of boscalid, fenpropidin, and fenpropimorph; in percent (*n*=3, error bars equal standard deviation; green area represents acceptable comparability from 80 to 120%)

The results of quantification with both methods were compared. Figure 4 shows the difference between the methods, expressed as the concentration determined with LC-MS/MS divided by the concentration determined with GC-MS/MS, in percent. The average difference for all three analytes (97% for boscalid, 104% for fenpropimorph, and 114% for fludioxonil) laid well within the desired range of 80–100%, underlining the comparability of results from quantification of the same samples with the two different methods.

In the following, the three bat guano samples were analysed with LC-MS/MS (Table 2). This approach enabled the quantification of fenpropimorph in the samples from June and July (0.5 µg kg^−1^ each) and of fluopyram in the sample from July (1.0 µg kg^−1^) even below the LLOQ (2.5 µg kg^−1^) of the GC-MS/MS method. The lower LLOQs of some pesticides in the LC-MS/MS method provide a valuable complement regarding analytes that are easier assessible with LC methods than with GC methods. The measured concentration of fludioxonil differs with 67 µg kg^−1^ from the result of the GC-MS/MS analysis (85 µg kg^−1^). However, regarding the differences of the two methods, applying different measurement techniques, operators, and standards, a deviation of 21% when quantifying with the two approaches is acceptable and underlines the plausibility of the results. Interestingly, no other pesticides of the 97 current used pesticides from the LC-MS/MS method were detected. This may be due to the small set of only three samples as well as to the composition of the matrix. In mammals, faeces are generally a lipophilic matrix and therefore preferably used for analysis of lipophilic compounds, such as POPs and PAHs, as opposed to hydrophilic matrices like urine [61].

## Conclusions

Within this work, an analytical method for the parallel quantification of 119 analytes in bat guano was developed and validated. The method is based on the QuEChERS sample preparation [42], presenting a modified approach of the DIN EN 15662 [43]. Validation was performed according to SANTE/11312/2021 [44] regarding linearity and lower limit of quantification, recovery, and precision. The developed methodology provided sufficient linearity and recovery with a high precision. Thus, the method allows for the rapid and precise parallel quantification of not only pesticides and POPs, but also a variety of other potential contaminants, such as flame retardants, plasticizers, UV blockers, hormones, and APIs. The presented method provides an important tool for non-invasive contamination monitoring of bat populations, facilitating the monitoring of contamination changes over time and paving the way for a better understanding of the effects different classes of pollutants may have on bat populations. The analysis of three guano samples collected in a nursery roost of *Myotis myotis* revealed the presence of 32 different analytes in the guano. Analysis of the same samples with an LC-MS/MS underlined the reliability of the obtained results and provided a tool to quantify individual analytes with a greater sensitivity. These results show that the developed methodology is suitable to quantify a variety of analytes in bat guano and it can be combined easily with an LC-MS/MS method. Both methods together cover 193 different lipophilic and hydrophilic pollutants. The results of this study also show that regarding pesticides mostly fungicides can contaminate bat populations, but that bats are already exposed to other potentially harmful substances such as plasticizers, flame retardants, or UV blockers. The effects of these substances on wildlife and especially on bats are mostly unknown. Further analyses are needed for a more precise interpretation of the results and conclusions on the effects of bat exposure.

### Supplementary Information

Below is the link to the electronic supplementary material.

Supplementary file1 (PDF 386 KB)

## Declarations

### Source of biological material

Bat guano was collected in a nursery roost of *Myotis myotis* in Ahrbrück, Germany, with permission of the Naturschutzbund Deutschland (NABU). Special attention was paid to ensuring that no bats were disturbed during sampling.
