# Supplementary material for: Multiresidue analysis of bat guano using GC-MS/MS
Source: Anal Bioanal Chem. 2024 Apr 2;416(13):3149–60. doi: 10.1007/s00216-024-05263-3 (PMC11068669; doi:10.1007/s00216-024-05263-3)
Supplement: Supplementary file 1 — Supplementary file1 (PDF 386 KB) [file 216_2024_5263_MOESM1_ESM.pdf]

Multiresidue analysis of bat guano using GC-MS/MS

Michelle Peter[1], Nikita Bakanov[2], Xenia Mathgen[3,4], Carsten A. Brühl[2], Michael Veith[3], and Christoph Müller[1*]

* corresponding author: Christoph Müller. E-mail: Christoph.mueller@cup.uni-muenchen.de; Tel: +49 89 2180 77250

[1] Department of Pharmacy, Center for Drug Research, Ludwig-Maximilians-Universität München, 81377 Munich, Germany

[2] iES Landau, Institute of Enivonmental Sciences Landau, University Kaiserslautern-Landau, 76829 Landau, Germany

[3] Department of Biogeography, Trier University, 54296 Trier, Germany

[4] State Office for Agriculture and Environement of Western Pomerania, 18439 Stralsund, Germany

Table S1 List of all analytes, including classification, retention time (RT), applied MRM transitions (quantifier in bold letters) and collision energy (CE); detected analytes highlighted in black; API active pharmaceutical ingredients, PAH polycyclic aromatic hydrocarbon, POP persistent organic pollutants; [1] analytes with two or three isomers, all retention times given

Analyte	Classification	RT [min]	Precursor ion	Product ion	CE [eV]
Acenaphthene	PAH	5.98	153.0	126.0	35
			**153.0**	**77.0**	35
			153.0	51.0	45
Acenaphthylene	PAH	5.70	**152.0**	**126.0**	20
			152.0	102.1	25
			152.0	76.0	35
Acetaminophen	API (analgesic)	7.45	151.0	109.1	5
			**151.0**	**80.0**	25
			109.0	80.0	15
Ametoctradin	pesticide (fungicide)	14.91	275.0	246.2	0
			275.0	190.3	15
			**246.0**	**188.2**	25
Atrazine	pesticide (herbicide)	7.64	215.0	200.1	5
			**215.0**	**58.1**	10
			200.0	94.1	15
Avobenzone	UV blocker	15.87	310.1	295.2	10
			310.1	135.1	25
			**310.1**	**108.1**	10
Azoxystrobin	pesticide (fungicide)	18.00	344.1	182.9	25
			**344.1**	**171.9**	40
			344.1	155.8	40
Azoxystrobin-d_4	internal standard	17.99	407.0	348.0	5
			392.0	364.0	5

Analyte	Classification	RT [min]	Precursor ion	Product ion	CE [eV]
			**348.0**	**172.1**	35
BDE 47	POP	13.98	485.7	325.7	20
			325.8	216.8	30
			**325.8**	**137.9**	50
BDE 99	POP	15.91	565.6	405.6	20
			**563.6**	**403.7**	20
			403.7	296.7	30
Benzophenone-1	UV blocker	10.66	**213.0**	**128.1**	25
			213.0	77.0	40
			213.0	51.0	45
BHC-β	POP	7.74	218.9	183.1	5
			216.9	181.1	5
			**181.0**	**145.0**	15
BHC-γ/Lindane	POP	7.84	218.9	183.1	5
			216.9	181.0	5
			**181.0**	**145.0**	15
BHT	antioxidant	5.92	220.2	205.2	10
			205.2	177.1	5
			**205.2**	**57.1**	10
Bifenazate	pesticide (acaricide)	13.60	184.1	91.1	40
			184.1	77.0	40
			**168.1**	**140.1**	10
Bifenthrin	pesticide	13.55	**181.2**	**165.2**	25

Analyte	Classification	RT [min]	Precursor ion	Product ion	CE [eV]
	(insecticide)		181.0	115.1	45
			166.0	115.1	35
Boscalid	pesticide (fungicide)	16.20	**140.0**	**112.0**	10
			140.0	76.0	25
			111.9	76.0	15
Caffeine	indicator for human pollution	8.51	194.0	109.1	10
			**194.0**	**55.0**	20
			109.0	55.0	5
Carbamazepine[1]	API (anticonvulsant)	12.80	**236.0**	**193.1**	5
			193.0	165.1	20
			193.0	139.1	40
		9.59	**193.0**	**165.1**	20
			193.0	89.0	45
			192.0	165.1	10
Carbetamide	pesticide (herbicide)	9.70	120.1	77.0	15
			119.1	91.0	15
			**119.1**	**64.1**	25
Carbofuran	pesticide (insecticide)	7.56	164.0	149.1	5
			**164.0**	**103.1**	20
			149.0	121.1	5
Celestolide	fragrance	7.38	**244.2**	**229.2**	5
			229.2	173.1	0
			229.2	57.1	10
Chlordane-*cis*	POP	10.91	374.8	265.8	15
			**372.8**	**265.8**	15
			271.7	236.9	15
Chlordane-*trans*	POP	10.65	374.8	265.8	15
			**372.8**	**265.8**	15
			271.7	236.9	15
Chlorpyrifos	pesticide (insecticide)	9.60	313.8	257.8	15
			**196.9**	**107.0**	40
			196.9	98.0	30
Clorpyrifos-d_{10}	internal standard	9.54	325.9	262.1	10
			**323.9**	**260.0**	10
			259.8	167.0	15
Chlorpyrifos-methyl	pesticide (insecticide)	8.80	287.9	92.9	20
			285.9	93.0	25
			**124.9**	**47.0**	15
Climbazole	API (antimycotic)	10.99	**206.9**	**111.0**	20
			206.9	75.0	35
			180.0	125.0	5
Clopidogrel	API (antiaggregant)	12.93	262.0	152.0	10
			**262.0**	**125.0**	25
			262.0	89.0	45
Clotrimazole	API (antimycotic)	10.99	278.1	243.1	5
			**278.1**	**165.1**	25
			243.1	165.1	15
Codeine	API (analgesic)	13.34	299.1	214.1	25
			**299.1**	**162.1**	5
			229.0	214.1	5
(-)-Cotinine	indicator for human pollution (metabolite)	7.59	**176.1**	**98.1**	5
			176.1	69.0	5
			98.1	54.0	30

Analyte	Classification	RT [min]	Precursor ion	Product ion	CE [eV]
Cybutryne	pesticide (fungicide)	10.36	253.0	196.1	15
			253.0	182.0	10
			**182.1**	**109.1**	5
Cyflufenamid	pesticide (fungicide)	11.63	412.0	294.9	5
			188.1	88.0	35
			**118.1**	**90.0**	10
Cyhalothrin-γ	pesticide (insecticide)	14.52	**208.0**	**181.0**	5
			208.0	152.0	25
			197.0	161.1	5
Cyhalothrin-λ	pesticide (insecticide)	14.33	**208.0**	**181.0**	5
			208.0	152.0	25
			197.0	161.1	5
Cypermethrin[1]	pesticide (insecticide)	16.12	165.0	127.1	0
		16.21	165.0	91.1	10
		16.29	**162.9**	**127.0**	0
DDE-*p,p'*	POP (metabolite)	11.25	317.8	248.0	15
			315.8	246.0	15
			**246.1**	**176.2**	30
DDT-*p,p'*	POP	12.55	237.0	165.2	20
			235.0	199.2	15
			**235.0**	**165.2**	20
DEHA	plasticizer	12.85	147.0	55.1	15
			**129.1**	**101.1**	0
			129.1	83.0	5
DEHTP	plasticizer	15.48	261.1	149.0	5
			167.0	79.0	10
			**149.0**	**65.0**	10
Deltamethrin	pesticide (insecticide)	17.73	**252.9**	**174.0**	0
			252.9	93.1	15
			251.0	172.0	0
Desmedipham	pesticide (herbicide)	7.36	181.0	122.0	10
			**181.0**	**109.0**	10
			122.0	94.0	10
Diazepam	API (anxiolytic)	11.34	256.1	221.2	5
			256.1	165.1	35
			**221.1**	**206.1**	35
Dicofol, *o, p'*-	POP	9.68	**139.0**	**111.0**	10
			139.0	75.0	30
			111.0	74.0	40
Dieldrin	POP	11.34	277.0	241.0	5
			**262.9**	**193.0**	35
			262.9	191.0	35
Difenoconazole[1]	pesticide (fungicide)	17.43	324.8	266.8	15
		17.50	**322.8**	**264.8**	15
			264.9	202.0	20
Diflubenzuron	pesticide (insecticide)	5.00	**157.0**	**141.0**	5
			141.0	113.0	10
			141.0	63.0	25
10,11-Dihydro-carbamazepine[1]	API (metabolite)	9.28	**195.0**	**180.1**	10
			194.0	179.0	15
			194.0	167.1	20
		12.48	**238.0**	**195.1**	5
			238.0	180.1	15
			194.0	179.1	15

Analyte	Classification	RT [min]	Precursor ion	Product ion	CE [eV]
Dimethomorph[1]	pesticide (fungicide)	18.06	302.9	164.9	10
		18.33	**300.9**	**165.0**	10
			300.9	138.8	15
Diphenylamine	industrial chemical	6.70	169.0	66.0	20
			**169.0**	**51.0**	45
			168.1	139.0	35
DPHP	plasticizer	16.11	**167.0**	**149.0**	0
			167.0	65.0	25
			149.0	65.0	20
EHS	UV blocker	8.04	138.0	120.0	0
			**120.1**	**92.0**	5
			120.1	63.0	30
Epoxiconazole	pesticide (fungicide)	13.23	**192.0**	**138.1**	10
			192.0	111.0	25
			138.0	75.0	25
17β-Estradiol	API (hormone)	14.95	272.1	213.1	10
			**272.1**	**172.1**	5
			213.0	133.1	10
Estrone	API (metabolite)	14.85	**270.1**	**185.1**	5
			270.1	172.1	10
			172.0	131.0	10
17α-Ethinylestradiol	API (estrogen medication)	15.48	**296.1**	**213.1**	10
			213.1	133.1	10
			160.0	127.0	20
Ethoxyquin	antioxidant	7.50	**202.0**	**174.1**	10
			202.0	130.1	35
			202.0	77.0	45
Fenazaquin	pesticide (acaricide)	13.84	**160.0**	**145.2**	5
			160.0	117.1	20
			146.0	118.1	10
Fenhexamid	pesticide (fungicide)	12.68	301.0	97.0	15
			179.0	115.0	15
			**177.1**	**113.0**	15
Fenpropidin	pesticide (fungicide)	9.18	**273.0**	**98.0**	5
			145.0	117.0	10
			117.0	91.0	15
Fenpropimorph	pesticide (fungicide)	9.57	128.1	110.1	5
			128.1	86.1	10
			**128.1**	**70.1**	10
Fenvalerate	pesticide (insecticide)	17.21	419.1	166.8	10
			**167.0**	**125.1**	10
			167.0	89.0	40
Fipronil	pesticide (biocide)	10.35	368.8	214.8	25
			**366.8**	**212.8**	25
			350.8	254.8	15
Fipronil sulfone	pesticide (metabolite)	11.48	384.8	256.8	20
			**382.8**	**254.9**	20
			254.9	227.9	15
Fluazifop-P-butyl	pesticide (herbicide)	11.64	382.9	282.0	10
			**281.9**	**238.0**	15
			254.0	146.1	15
Fludioxonil	pesticide (fungicide)	11.24	248.0	182.1	10
			248.0	154.1	20
			**248.0**	**127.1**	30

Analyte	Classification	RT [min]	Precursor ion	Product ion	CE [eV]
Fluopyram	pesticide (fungicide)	10.32	395.9	223.1	5
			**222.9**	**196.0**	10
			222.9	187.1	10
Fluoranthene	PAH	10.34	202.0	176.0	25
			202.0	150.1	45
			**101.0**	**88.0**	5
Fluorene	PAH	6.53	166.0	115.0	35
			**165.0**	**115.1**	25
			165.0	63.0	45
Flupyradifurone	pesticide (insecticide)	14.58	288.0	126.1	15
			128.0	90.0	10
			**126.0**	**73.0**	25
Fluvalinate-*tau*[1]	pesticide (insecticide)	17.19	252.0	200.0	15
		17.25	**250.0**	**200.1**	15
			250.0	198.1	40
Galaxolide	fragrance	8.49	**243.0**	**213.2**	5
			243.0	171.1	10
			243.0	155.1	35
Heptachlor	POP	8.98	273.7	238.9	15
			273.7	236.9	15
			**271.7**	**236.9**	15
Imidacloprid	pesticide (insecticide)	11.01	**211.0**	**113.0**	15
			126.0	89.9	5
			126.0	73.0	25
Isoproturon	pesticide (herbicide)	4.46	161.1	146.1	5
			**146.2**	**128.1**	5
			146.2	77.0	20

Analyte	Classification	RT [min]	Precursor ion	Product ion	CE [eV]
Lenacil	pesticide (herbicide)	12.66	233.9	153.1	5
			153.1	110.1	20
			**153.1**	**82.1**	20
Lilial	fragrance	6.04	189.0	131.1	5
			189.0	115.1	35
			**189.0**	**91.1**	20
4-MBC	UV blocker	10.22	211.0	169.1	5
			171.0	143.1	0
			**171.0**	**128.1**	15
Mestranol	API (estrogen medication)	15.09	**310.2**	**227.1**	10
			227.2	171.1	10
			227.2	147.1	10
Metamitron	pesticide (herbicide)	11.55	202.1	186.1	5
			**202.1**	**104.1**	15
			173.1	132.1	10
Metazachlor	pesticide (herbicide)	10.19	209.0	133.2	10
			**209.0**	**132.2**	15
			209.0	117.1	35
Metrafenone	pesticide (fungicide)	14.96	**394.8**	**364.8**	15
			376.9	346.8	20
			226.9	169.0	10
Miconazole	API (antimycotic)	17.64	**159.0**	**123.0**	15
			159.0	89.0	25
			159.0	63.0	45
Myclobutanil	pesticide (fungicide)	11.42	**179.0**	**125.1**	10
			179.0	90.0	30
			150.0	123.0	15

Analyte	Classification	RT [min]	Precursor ion	Product ion	CE [eV]
Octocrylene	UV blocker	15.03	249.1	204.1	5
			249.1	165.1	30
			**204.1**	**176.0**	25
4-*tert*-Octylphenol	industrial chemical	6.54	**135.0**	**107.1**	10
			135.0	77.0	20
			135.0	51.0	40
OMC	UV blocker	12.28	**178.0**	**161.1**	10
			178.0	89.0	35
			161.0	89.0	35
Oxybenzone	UV blocker	9.98	228.0	184.0	25
			**227.0**	**184.0**	20
			227.0	128.1	35
PCB 101	POP	10.75	327.9	255.9	30
			**325.9**	**255.9**	30
			253.9	184.0	35
PCB 138	POP	12.74	361.9	289.9	30
			**359.9**	**289.9**	30
			287.9	217.9	40
PCB 153	POP	12.24	361.9	289.9	25
			**359.9**	**289.9**	25
			287.9	217.9	40
PCB 180	POP	13.91	395.8	325.8	30
			393.8	358.8	15
			**393.8**	**323.8**	30
PCB 28	POP	8.70	258.0	186.0	25
			**256.0**	**186.0**	25
			186.0	151.0	25

Analyte	Classification	RT [min]	Precursor ion	Product ion	CE [eV]
PCB 52	POP	9.26	291.9	221.9	25
			**289.9**	**219.9**	25
			255.0	220.0	10
Pentachloronitrobenzene	pesticide (fungicide)	7.91	294.8	236.8	15
			248.8	213.8	15
			**141.9**	**106.9**	30
Permethrin[1]	pesticide (insecticide)	15.23	165.0	91.0	15
		15.36	**163.0**	**127.0**	5
			127.0	91.0	10
Picolinafen	pesticide (herbicide)	13.59	376.0	239.1	10
			**376.0**	**238.1**	20
			238.1	145.1	25
Pirimicarb	pesticide (insecticide)	8.42	**238.0**	**166.2**	10
			166.0	71.1	25
			166.0	55.1	20
Propiconazole[1]	pesticide (fungicide)	12.59	258.8	172.9	15
		12.71	172.9	109.0	30
			**172.9**	**74.0**	45
Pyrene	PAH	10.80	202.0	174.1	45
			202.0	151.1	40
			**101.0**	**88.0**	5
Pyrene-d_{10}	internal standard	10.80	**212.0**	**206.6**	30
			212.0	178.8	45
			106.0	91.8	5
Pyrimethanil	pesticide (fungicide)	8.00	198.0	183.1	15
			198.0	158.1	20
			**198.0**	**118.1**	35

Analyte	Classification	RT [min]	Precursor ion	Product ion	CE [eV]
Quinoxyfen	pesticide (fungicide)	12.55	306.8	237.0	20
			271.9	237.1	10
			**237.0**	**208.1**	30
Simazine	pesticide (herbicide)	7.59	201.0	186.0	5
			**201.0**	**173.0**	0
			186.0	91.0	5
Spirodiclofen	pesticide (acaricide)	15.27	**312.1**	**259.0**	10
			312.1	108.9	15
			157.0	73.0	25
TDCPP	flame retardant	12.48	381.0	158.9	5
			**209.0**	**99.0**	5
			191.0	74.9	5
Tebuconazole	pesticide (fungicide)	12.93	250.0	125.0	20
			125.0	99.0	20
			**125.0**	**89.0**	15
Terbuthylazine	pesticide (herbicide)	7.85	**229.0**	**173.0**	0
			214.0	104.0	20
			214.0	71.0	20
Terbuthylazine-desethyl	pesticide (metabolite)	7.12	**186.2**	**104.0**	15
			145.1	110.1	10
			145.1	68.1	20
Terbutryn	pesticide (herbicide)	9.29	**241.2**	**185.1**	0
			241.2	170.0	10
			185.1	170.0	0
Testosterone	API (hormone)	15.19	**288.2**	**124.1**	5
			288.2	109.1	20
			246.1	185.1	5

Analyte	Classification	RT [min]	Precursor ion	Product ion	CE [eV]
Tetraconazole	pesticide (fungicide)	9.76	336.0	217.9	20
			**336.0**	**203.8**	30
			170.9	136.0	10
Thiabendazole	pesticide (fungicide)	10.41	201.0	174.0	15
			201.0	130.0	30
			**173.9**	**65.0**	30
Tolclofos-methyl	pesticide (fungicide)	8.89	**267.0**	**252.0**	15
			267.0	93.0	30
			267.0	63.0	45
Tonalid[1]	fragrance	8.49	**258.0**	**243.2**	0
		8.57	243.0	187.1	0
			243.0	57.1	10
Torasemide	API (diuretic)	14.24	263.0	246.1	0
			**263.0**	**181.1**	20
			181.0	128.0	15
Triclosan	API (disinfectant)	10.61	287.9	218.0	15
			287.9	145.9	15
			**145.9**	**111.0**	15
Triphenyl phosphate	flame retardant	13.00	**326.0**	**325.0**	5
			325.0	169.1	20
			325.0	77.0	35
Uvinul A Plus	UV blocker	18.17	**397.0**	**382.2**	10
			382.0	280.1	10
			382.0	149.0	15
Verapamil	API (antiarrhythmic)	19.13	303.1	260.2	5
			303.1	151.1	15
			**303.1**	**58.0**	20

**Analyte**	**Classification**	**RT [min]**	**Precursor ion**	**Product ion**	**CE [eV]**
Zoxamide	pesticide (fungicide)	13.19	259.9	189.0	10
			**257.9**	**187.1**	10
			189.0	161.1	15

Table S2 Summary of validation data, including LLOQ, R^2, recovery, method precision and injection precision; tested concentrations for medium=500 µg kg^{-1} and high=1000 µg $^{-1}$ (for the two PAHs 2000 µg kg^{-1} and 3500 µg kg^{-1}) (n=6 for all experiments); detected analytes highlighted in black; [1] analyte with two or three isomers

**Analyte**	**Linearity**		**Recovery**	**Precision data at low, medium, and high concentration**					
				Low Concentration		Medium Concentration		High Concentration	
	Linear range [µg kg^{-1}]	R^2	Mean Recovery [%]	Method precision RSD [%]	Injection precision RSD [%]	Method precision RSD [%]	Injection precision RSD [%]	Method precision RSD [%]	Injection precision RSD [%]
Acenaphthene	100 – 1250	0.999	95.1	5.2	2.3	6.7	3.9	6.0	3.2
Acenaphthylene	100 – 1250	0.998	96.6	4.9	1.7	5.0	3.1	4.5	2.0
Acetaminophen	100 – 1250	0.993	99.6	12.0	6.0	10.3	4.8	6.2	7.0
Ametoctradin	100 – 1250	0.993	102.5	8.6	3.7	5.4	2.2	4.4	3.3
Atrazine	2.5 – 1250	0.997	100.6	12.5	3.5	6.4	1.3	14.0	1.2
Avobenzone	50 – 1250	0.995	89.5	10.7	2.6	8.4	1.3	9.4	3.4
Azoxystrobin	10 – 1250	0.999	97.6	9.0	5.8	5.8	2.0	7.7	2.5
BDE 47	5.0 – 1250	0.989	86.4	13.4	6.2	5.7	3.2	9.0	2.6
BDE 99	2.5 – 1250	0.991	57.3	19.4	11.3	10.9	1.9	4.5	2.9
Benzophenone-1	50 – 1250	0.995	110.4	4.4	3.2	3.8	3.1	6.5	4.4
BHC-β	2.5 – 1250	0.999	106.2	8.5	6.3	6.1	1.7	16.8	1.7
BHC-γ/Lindane	2.5 – 1250	0.999	102.2	11.1	6.7	5.9	1.2	18.4	2.0
BHT	50 – 1250	0.999	92.9	13.9	4.2	9.0	6.8	4.1	4.4
Bifenazate	250 – 1250	0.974	131.6	15.1	5.2	6.2	4.9	3.8	7.9
Bifenthrin	5.0 – 1250	0.996	112.4	13.2	5.9	5.2	3.9	6.1	2.7
Boscalid	5.0 – 1250	0.993	113.3	14.2	1.8	11.5	1.8	4.1	2.7
Caffeine	5.0 – 1250	0.996	83.6	7.4	3.6	3.7	1.4	3.8	1.6
Carbamazepine[1]	50 – 1250	0.978	92.9	15.1	2.1	9.2	3.5	7.7	2.3
Carbetamide	50 – 1250	0.994	97.9	17.9	7.5	5.0	8.0	4.2	7.5

**Analyte**	**Linearity**		**Recovery**	**Precision data at low, medium, and high concentration**					
				Low Concentration		Medium Concentration		High Concentration	
	Linear range [µg kg^{-1}]	R^2	Mean Recovery [%]	Method precision RSD [%]	Injection precision RSD [%]	Method precision RSD [%]	Injection precision RSD [%]	Method precision RSD [%]	Injection precision RSD [%]
Carbofuran	5.0 – 1250	0.997	97.8	14.0	5.7	3.8	7.9	9.1	8.1
Celestolide	5.0 – 1250	0.997	104.5	5.3	7.0	5.2	1.8	8.1	1.6
Chlordane-*cis*	5.0 – 1250	0.994	106.6	9.6	7.5	5.5	4.0	8.0	2.7
Chlordane-*trans*	5.0 – 1250	0.992	100.9	10.4	3.1	5.6	3.7	7.6	2.5
Chlorpyrifos	2.5 – 1250	0.997	101.8	19.3	6.3	3.8	0.7	4.3	1.4
Chlorpyrifos-methyl	2.5 – 1250	0.996	104.6	14.3	6.8	4.8	1.7	6.9	1.5
Climbazole	5.0 – 1250	0.996	118.5	13.4	2.4	6.5	3.4	6.2	3.4
Clopidogrel	2.5 – 1250	0.993	115.7	1.8	2.5	9.1	3.7	5.6	2.7
Clotrimazole	5.0 – 1250	0.997	119.9	6.8	6.5	7.7	2.7	6.7	3.2
Codeine	5.0 – 1250	0.993	81.5	13.7	7.9	7.5	3.6	10.5	2.9
(-)-Cotinine	10 – 1250	0.996	66.5	9.9	3.2	4.0	2.5	13.0	1.5
Cybutryne	5.0 – 1250	0.998	112.2	19.3	4.0	6.2	3.1	5.9	3.0
Cyflufenamid	50 – 1250	0.995	130.8	9.7	3.7	6.2	4.1	6.7	1.9
Cyhalothrin-γ	5.0 – 1250	0.995	100.7	14.2	3.9	8.3	3.1	6.0	2.3
Cyhalothrin-λ	100 – 1250	0.972	131.9	7.0	5.3	7.6	6.7	12.1	8.3
Cypermethrin[1]	5.0 – 1250	0.994	104.8	15.3	5.4	11.6	2.9	3.5	1.9
DDE-*p,p'*	5.0 – 1250	0.997	98.0	8.0	3.3	6.1	3.7	6.5	2.6
DDT-*p,p'*	5.0 – 1250	0.978	107.7	7.1	9.5	6.1	13.2	9.5	7.5
DEHA	50 – 1250	0.980	99.6	9.7	3.2	5.7	3.6	9.2	2.7
DEHTP	50 – 1250	0.994	46.1	6.8	3.4	11.9	1.9	9.7	3.8
Deltamethrin	10 – 1250	0.994	88.0	8.2	7.9	12.5	3.5	5.7	3.6
Desmedipham	50 – 1250	0.995	97.5	15.3	7.8	4.5	3.9	7.1	3.9

Analyte	Linearity		Recovery	Precision data at low, medium, and high concentration					
				Low Concentration		Medium Concentration		High Concentration	
	Linear range [µg kg^{-1}]	R^2	Mean Recovery [%]	Method precision RSD [%]	Injection precision RSD [%]	Method precision RSD [%]	Injection precision RSD [%]	Method precision RSD [%]	Injection precision RSD [%]
Diazepam	50 – 1250	0.991	109.7	18.2	3.7	6.8	3.7	4.9	2.8
Dicofol, *o, p'*-	2.5 – 1250	0.996	106.8	18.1	12.9	4.3	2.5	6.9	1.2
Dieldrin	50 – 1250	0.994	117.7	9.9	4.4	7.9	2.6	5.7	3.1
Difenoconazole[1]	5.0 – 1250	0.995	102.2	8.1	5.5	7.8	2.6	7.5	4.0
Diflubenzuron	5.0 – 1250	0.995	117.0	14.8	13.0	5.3	2.3	7.1	1.6
10,11-Dihydrocarbamazepine[1]	50 – 1250	0.980	79.9	11.2	3.6	9.4	2.3	6.3	1.4
Dimethomorph[1]	5.0 – 1250	0.998	101.7	11.0	16.1	7.4	2.4	7.4	3.4
Diphenylamine	50 – 1250	0.999	110.3	10.4	2.1	3.9	1.8	6.1	1.6
DPHP	50 – 1250	0.983	48.3	11.6	2.2	15.6	2.0	5.5	3.7
EHS	100 – 1250	0.985	100.4	8.1	2.2	3.7	1.4	8.2	1.5
Epoxiconazole	2.5 – 1250	0.995	118.4	4.9	3.7	7.1	3.7	9.1	2.9
17β-Estradiol	10 – 1250	0.997	102.8	18.4	7.1	9.3	1.8	6.5	3.6
Estrone	100 – 1250	0.999	102.4	6.7	4.0	10.6	1.9	9.2	3.4
17α-Ethinylestradiol	10 – 1250	0.996	103.3	13.2	3.4	7.0	2.0	11.8	3.0
Ethoxyquin	2.5 – 1250	0.997	87.5	9.1	7.5	9.2	3.6	9.1	2.9
Fenazaquin	250 – 1250	0.942	114.4	13.0	3.7	8.5	1.7	7.6	2.4
Fenhexamid	10 – 1250	0.999	117.4	10.6	12.2	5.9	3.3	8.5	3.4
Fenpropidin	2.5 – 1250	0.993	96.4	13.8	4.0	5.1	1.2	4.1	1.4
Fenpropimorph	2.5 – 1250	0.995	109.5	14.1	4.4	4.6	1.2	6.3	1.3
Fenvalerate	2.5 – 1250	0.996	92.5	17.6	9.2	11.3	2.7	6.1	1.8
Fipronil	2.5 – 1250	0.989	112.3	7.5	5.4	7.1	3.8	4.5	3.4
Fipronil sulfone	10 – 1250	0.986	117.1	18.5	2.6	9.2	3.5	8.3	3.0

Analyte	Linearity		Recovery	Precision data at low, medium, and high concentration					
				Low Concentration		Medium Concentration		High Concentration	
	Linear range [µg kg^{-1}]	R^2	Mean Recovery [%]	Method precision RSD [%]	Injection precision RSD [%]	Method precision RSD [%]	Injection precision RSD [%]	Method precision RSD [%]	Injection precision RSD [%]
Fluazifop-P-butyl	5.0 – 1250	0.994	113.6	11.1	5.1	9.4	3.7	5.9	2.7
**Fludioxonil**	**5.0 – 1250**	**0.996**	**115.7**	**9.3**	**5.2**	**10.3**	**3.6**	**6.0**	**2.9**
**Fluopyram**	**2.5 – 1250**	**0.996**	**120.0**	**11.3**	**8.7**	**7.2**	**4.1**	**4.4**	**3.4**
**Fluoranthene**	**750 – 5000**	**0.963**	**98.5**	**8.3**	**3.7**	**10.4**	**4.5**	**10.3**	**2.5**
**Fluorene**	**100 – 1250**	**0.998**	**102.2**	**11.0**	**3.0**	**4.7**	**1.1**	**10.0**	**2.1**
Flupyradifuron	10 – 1250	0.991	113.1	14.5	10.4	11.3	1.3	7.4	3.1
Fluvalinat-*tau*[1]	2.5 – 1250	0.999	109.4	15.8	3.7	8.7	1.9	6.1	3.2
**Galaxolide**	**50 – 1250**	**0.999**	**97.3**	**8.2**	**1.1**	**3.8**	**1.4**	**5.3**	**1.8**
Heptachlor	2.5 – 1250	0.998	104.5	10.7	7.4	4.3	4.2	5.8	2.8
Imidacloprid	50 – 1250	0.995	135.4	19.5	8.0	11.2	3.8	7.2	4.8
Isoproturon	2.5 – 1250	0.996	106.2	15.3	2.4	4.5	1.0	7.4	0.6
Lenacil	10 – 1250	0.995	118.8	7.2	4.0	8.3	4.1	8.8	3.0
Lilial	5.0 – 1250	0.990	92.4	11.2	4.9	9.8	2.9	6.2	1.5
4-MBC	100 – 1250	0.994	108.9	14.0	3.5	5.2	3.6	7.1	2.3
Mestranol	5.0 – 1250	0.997	101.8	12.8	5.0	8.9	2.0	6.8	3.2
Metamitron	50 – 1250	0.990	98.4	17.8	7.9	7.1	6.3	6.2	3.6
Metazachlor	5.0 – 1250	0.995	106.8	3.5	7.8	5.3	4.0	5.0	3.1
Metrafenone	50 – 1250	0.987	100.6	17.8	7.0	9.5	2.5	6.6	3.1
Miconazol	10 – 1250	0.994	106.3	7.0	9.9	11.2	1.7	6.1	3.7
Myclobutanil	2.5 – 1250	0.995	115.2	14.4	8.2	5.8	3.5	7.7	3.0
**Octocrylene**	**10 – 1250**	**0.995**	**106.9**	**13.9**	**4.4**	**9.0**	**1.5**	**7.5**	**3.3**
4-*tert*-Octylphenol	100 – 1250	0.992	109.3	7.1	1.8	4.1	1.9	7.2	2.0

**Analyte**	**Linearity**		**Recovery**	**Precision data at low, medium, and high concentration**					
				Low Concentration		Medium Concentration		High Concentration	
	Linear range [µg kg^{-1}]	R^2	Mean Recovery [%]	Method precision RSD [%]	Injection precision RSD [%]	Method precision RSD [%]	Injection precision RSD [%]	Method precision RSD [%]	Injection precision RSD [%]
OMC	5.0 – 1250	0.995	113.2	13.1	4.6	6.4	3.8	8.2	2.9
Oxybenzone	2.5 – 1250	0.995	115.7	12.4	3.8	7.3	3.4	6.9	3.0
PCB 101	2.5 – 1250	0.998	88.5	6.7	14.7	3.0	3.5	6.0	2.2
PCB 138	2.5 – 1250	0.973	73.0	19.2	6.9	4.1	4.7	11.0	2.4
PCB 153	2.5 – 1250	0.992	70.0	10.3	7.5	5.5	3.9	4.4	2.4
PCB 180	5.0 – 1250	0.992	54.5	9.9	8.3	5.9	3.5	7.3	2.2
PCB 28	2.5 – 1250	0.986	98.5	8.8	8.2	4.3	1.3	8.3	1.3
PCB 52	2.5 – 1250	0.992	98.3	12.3	16.0	4.3	1.6	8.3	2.1
Pentachloronitrobenzene	5.0 – 1250	0.996	98.8	16.4	9.1	5.7	1.3	10.7	1.0
Permethrin[1]	50 – 1250	0.997	106.9	11.9	8.4	11.8	1.5	9.5	3.1
Picolinafen	2.5 – 1250	0.984	100.4	8.9	3.8	6.2	4.1	4.6	2.2
Pirimicarb	2.5 – 1250	0.997	95.9	12.2	4.8	6.9	2.1	5.6	1.9
Propiconazole[1]	2.5 – 1250	0.936	114.4	13.6	8.0	6.6	3.6	7.6	3.1
Pyrene	750 – 5000	0.974	96.5	6.0	4.7	7.4	4.5	10.0	2.9
Pyrimethanil	2.5 – 1250	0.997	96.6	13.8	4.2	5.2	1.5	7.7	1.6
Quinoxyfen	2.5 – 1250	0.995	104.5	14.2	5.8	6.1	3.7	7.3	2.6
Simazine	50 – 1250	0.996	103.8	11.1	4.7	5.4	2.6	14.8	1.8
Spirodiclofen	50 – 1250	0.977	65.3	15.5	10.9	13	5.7	11.9	2.9
TDCPP	5.0 – 1250	0.992	81.8	16.2	6.6	8.4	3.9	6.5	2.4
Tebuconazole	2.5 – 1250	0.995	123.2	4.9	5.5	6.9	3.7	6.1	2.7
Terbuthylazine	5.0 – 1250	0.998	106.8	19.5	7.3	4.7	1.5	17.8	1.4
Terbuthylazine-desethyl	5.0 – 1250	0.995	110.2	6.5	5.4	4.1	1.3	8.3	1.3

Analyte	Linearity		Recovery	Precision data at low, medium, and high concentration					
				Low Concentration		Medium Concentration		High Concentration	
	Linear range [µg kg^{-1}]	R^2	Mean Recovery [%]	Method precision RSD [%]	Injection precision RSD [%]	Method precision RSD [%]	Injection precision RSD [%]	Method precision RSD [%]	Injection precision RSD [%]
Terbutryn	5.0 – 1250	0.996	102.6	5.4	4.9	5.3	1.3	7.6	1.5
Testosterone	100 – 1250	0.999	97.8	14.6	3.8	9.3	2.2	10.8	2.1
Tetraconazole	2.5 – 1250	0.998	99.7	7.8	1.9	4.9	1.3	7.3	1.6
Thiabendazole	10 – 1250	0.996	97.5	12.3	9.3	7.3	4.8	9.9	3.6
Tolclofos-methyl	5.0 – 1250	0.997	100.0	14.7	13.3	4.9	1.3	7.6	0.7
Tonalid[1]	10 – 1250	0.999	101.1	9.6	1.6	2.4	1.8	6.6	1.7
Torasemide	100 – 1250	0.973	92.2	13.0	6.4	13.0	5.0	12.8	8.5
Triclosan	2.5 – 1250	0.998	108.8	5.0	8.6	4.8	3.7	6.2	3.2
Triphenylphosphat	5.0 – 1250	0.995	111.2	14.2	5.4	8.9	3.8	5.9	2.6
Uvinul A Plus	5.0 – 1250	0.998	100.2	19.7	10.8	7.8	3.3	10.0	3.2
Verapamil	2.5 – 1250	0.996	108.8	7.8	5.9	9.1	2.3	6.7	5.3
Zoxamide	10 – 1250	0.973	87.7	16.0	6.8	14.7	12.7	8.4	8.4

Table S3 Summary of compounds with R^2 <0.990 and/or with a recovery outside the range of 70% to 120% from Table S2; "outliers" in bold; detected analytes highlighted in black; [1] analyte with two or three isomers

**Analyte**	**Linearity**		**Recovery**	**Precision data at low, medium, and high concentration**					
				Low Concentration		Medium Concentration		High Concentration	
	Linear range [µg kg^{-1}]	R^2	Mean Recovery [%]	Method precision RSD [%]	Injection precision RSD [%]	Method precision RSD [%]	Injection precision RSD [%]	Method precision RSD [%]	Injection precision RSD [%]
BDE 47	5.0 – 1250	**0.989**	86.4	13.4	6.2	5.7	3.2	9.0	2.6
BDE 99	2.5 – 1250	0.991	**57.3**	19.4	11.3	10.9	1.9	4.5	2.9
Bifenazate	250 – 1250	**0.974**	**131.6**	15.1	5.2	6.2	4.9	3.8	7.9
Carbamazepine[1]	50 – 1250	**0.978**	92.9	15.1	2.1	9.2	3.5	7.7	2.3
(-)-Cotinine	10 – 1250	0.996	**66.5**	9.9	3.2	4.0	2.5	13.0	1.5
Cyflufenamid	50 – 1250	0.995	**130.8**	9.7	3.7	6.2	4.1	6.7	1.9
Cyhalothrin-λ	100 – 1250	**0.972**	**131.9**	7.0	5.3	7.6	6.7	12.1	8.3
DDT-*p,p'*	5.0 – 1250	**0.978**	107.7	7.1	9.5	6.1	13.2	9.5	7.5
DEHA	50 – 1250	**0.980**	99.6	9.7	3.2	5.7	3.6	9.2	2.7
DEHTP	50 – 1250	0.994	**46.1**	6.8	3.4	11.9	1.9	9.7	3.8
10,11-Dihydrocarbamazepine[1]	50 – 1250	**0.980**	**79.9**	11.2	3.6	9.4	2.3	6.3	1.4
DPHP	50 – 1250	**0.983**	**48.3**	11.6	2.2	15.6	2.0	5.5	3.7
Fenazaquin	250 – 1250	**0.942**	114.4	13.0	3.7	8.5	1.7	7.6	2.4
Fipronil sulfone	10 – 1250	**0.986**	117.1	18.5	2.6	9.2	3.5	8.3	3.0
Fluoranthene	750 – 5000	**0.963**	98.5	8.3	3.7	10.4	4.5	10.3	2.5
Imidacloprid	50 – 1250	0.995	**135.4**	19.5	8.0	11.2	3.8	7.2	4.8
Metrafenone	50 – 1250	**0.987**	100.6	17.8	7.0	9.5	2.5	6.6	3.1
PCB 138	2.5 – 1250	**0.973**	**73.0**	19.2	6.9	4.1	4.7	11.0	2.4
PCB 180	5.0 – 1250	0.992	**54.5**	9.9	8.3	5.9	3.5	7.3	2.2
PCB 28	2.5 – 1250	**0.986**	98.5	8.8	8.2	4.3	1.3	8.3	1.3

**Analyte**	**Linearity**		**Recovery**	**Precision data at low, medium, and high concentration**					
				Low Concentration		Medium Concentration		High Concentration	
	Linear range [µg kg^{-1}]	R^2	Mean Recovery [%]	Method precision RSD [%]	Injection precision RSD [%]	Method precision RSD [%]	Injection precision RSD [%]	Method precision RSD [%]	Injection precision RSD [%]
Picolinafen	2.5 – 1250	**0.984**	100.4	8.9	3.8	6.2	4.1	4.6	2.2
Propiconazole[1]	2.5 – 1250	**0.936**	114.4	13.6	8.0	6.6	3.6	7.6	3.1
Pyrene	750 – 5000	**0.974**	96.5	6.0	4.7	7.4	4.5	10.0	2.9
Spirodiclofen	50 – 1250	**0.977**	**65.3**	15.5	10.9	13	5.7	11.9	2.9
Tebuconazole	2.5 – 1250	0.995	**123.2**	4.9	5.5	6.9	3.7	6.1	2.7
Torasemide	100 – 1250	**0.973**	92.2	13.0	6.4	13.0	5.0	12.8	8.5
Zoxamide	10 – 1250	**0.973**	87.7	16.0	6.8	14.7	12.7	8.4	8.4
